# A GFP Expressing Influenza A Virus to Report *In Vivo* Tropism and Protection by a Matrix Protein 2 Ectodomain-Specific Monoclonal Antibody

**DOI:** 10.1371/journal.pone.0121491

**Published:** 2015-03-27

**Authors:** Sarah De Baets, Judith Verhelst, Silvie Van den Hoecke, Anouk Smet, Michael Schotsaert, Emma R. Job, Kenny Roose, Bert Schepens, Walter Fiers, Xavier Saelens

**Affiliations:** 1 Department of Medical Protein Research, VIB, Ghent, Belgium; 2 Department of Biomedical Molecular Biology, Ghent University, Ghent, Belgium; Icahn School of Medicine at Mount Sinai, UNITED STATES

## Abstract

The severity of influenza-related illness is mediated by many factors, including *in vivo* cell tropism, timing and magnitude of the immune response, and presence of pre-existing immunity. A direct way to study cell tropism and virus spread *in vivo* is with an influenza virus expressing a reporter gene. However, reporter gene-expressing influenza viruses are often attenuated *in vivo* and may be genetically unstable. Here, we describe the generation of an influenza A virus expressing GFP from a tri-cistronic NS segment. To reduce the size of this engineered gene segment, we used a truncated NS1 protein of 73 amino acids combined with a heterologous dimerization domain to increase protein stability. GFP and nuclear export protein coding information were fused in frame with the truncated NS1 open reading frame and separated from each other by 2A self-processing sites. The resulting PR8-NS1(1–73)GFP virus was successfully rescued and replicated as efficiently as the parental PR8 virus *in vitro* and was slightly attenuated *in vivo*. Flow cytometry-based monitoring of cells isolated from PR8-NS1(1–73)GFP virus infected BALB/c mice revealed that GFP expression peaked on day two in all cell types tested. In particular respiratory epithelial cells and myeloid cells known to be involved in antigen presentation, including dendritic cells (CD11c^+^) and inflammatory monocytes (CD11b^+^ GR1^+^), became GFP positive following infection. Prophylactic treatment with anti-M2e monoclonal antibody or oseltamivir reduced GFP expression in all cell types studied, demonstrating the usefulness of this reporter virus to analyze the efficacy of antiviral treatments *in vivo*. Finally, deep sequencing analysis, serial *in vitro* passages and *ex vivo* analysis of PR8-NS1(1–73)GFP virus, indicate that this virus is genetically and phenotypically stable.

## Introduction

In humans, symptoms following infection with influenza A or B virus range from asymptomatic to very severe disease and even death. People are susceptible to influenza throughout life and have a higher risk of developing complications during early childhood and at later age (>65 years) [1–3]. The disease outcome is determined by many host factors, such as the timing and magnitude of the innate immune response, the level of pre-existing immunity, comorbidities, and genetic predisposition [1, 4–7]. Another important determinant of the severity of influenza-related disease is the *in vivo* cell tropism of the virus. In mice, for instance, highly pathogenic A/Puerto Rico/8/34 (PR8) and low pathogenic A/Texas/36/91 virus achieve similar infectious particle loads, but PR8 virus spreads better in lung tissue [8].

The host cell surface receptors for influenza viruses are oligosaccharides with a terminal sialic acid. These receptors, which are bound by the viral hemagglutinin (HA), are important determinants of influenza virus tropism and transmission [9–11]. In general, HA on human influenza viruses preferentially binds to sialic acid that is α2,6-linked to galactose, whereas HA expressed by avian influenza viruses prefers α2,3-linked sialic acid [12]. In addition to the specificity of HA, many other factors also determine the host range of influenza viruses, including the presence or absence of a polybasic cleavage site in HA, the efficiency of cell and nuclear entry, and viral genome replication [13, 14].

Relatively little is known about the *in vivo* cell tropism of influenza viruses and how pre-existing immunity or antivirals affect virus spread. Live imaging of virus-infected cells is a versatile way to study their subcellular behavior and *in vivo* tropism. For this purpose, viruses expressing green fluorescent protein (GFP) or luciferase have been generated and used [15–18]. For large DNA viruses and some RNA viruses such as members of the *Paramyxoviridae*, GFP-expressing viruses are often genetically stable and may retain their pathogenicity in laboratory animals [19, 20]. The generation of replication-competent influenza viruses expressing heterologous proteins poses several challenges: (i) the segmented genome of influenza viruses does not allow large insertions, (ii) insertion usually compromises virus fitness and *in vivo* pathogenicity, (iii) insertion of a reporter sequence could disrupt packaging sequences, which are present in both the coding and non-coding regions of each genome segment, and (iv) because all viral genes are essential for viral fitness, none of them can be replaced by a reporter gene without loss of multi-cycle replication [16, 21, 22]. Replication-competent GFP-expressing influenza viruses have been generated by inserting the GFP-coding sequence in the neuraminidase (NA), PA, or NS gene segment [16, 22–27]. Such viruses express GFP in infected cells *in vitro* as well as *in vivo*, and are useful tools for monitoring the course of an influenza virus infection in animal models and for screening of influenza antiviral drugs. However, they are somewhat attenuated *in vivo* and can lose GFP expression over time [16, 22, 24, 25].

Here, we report the construction of a GFP-expressing influenza virus, PR8-NS1(1–73)GFP, with a truncated NS1 open reading frame. This virus replicates as efficiently as wild type PR8 virus in MDCK cells. Deep sequencing analysis revealed that the parental PR8 virus and this novel GFP-expressing influenza A virus display a similar genetic homogeneity. As expected, truncation of the NS1 gene resulted in slight attenuation of PR8-NS1(1–73)GFP in laboratory mice compared to wild type PR8 virus. Finally, we demonstrate the usefulness of this PR8-NS1(1–73)GFP virus to study the viral cell tropism *ex vivo* and to evaluate the effects of treatment with oseltamivir and an anti-M2e monoclonal antibody on viral tropism.

## Materials and Methods

### Ethics statement

All mouse experiments were conducted according to national (Belgian Law 14/08/1986 and 22/12/2003, Belgian Royal Decree 06/04/2010) and European legislation (EU Directives 2010/63/EU, 86/609/EEG) on animal regulations. Experiments on mice were approved by the ethics committee of VIB (Vlaams Instituut voor Biotechnologie) site Ghent, Ghent University, Faculty of Sciences (Eth. Com. No. 2013-079) and efforts were made to avoid or diminish suffering of the animals. Before infection, mice were sedated with isoflurane or by intraperitoneal injection of ketamine (100 μg/g)/xylazine (10 μg/g). After infection, body weight was monitored for 14 days and mice were euthanized by cervical dislocation when they lost more than 25% of their initial body weight. To sample the lungs of infected mice, mice were terminally sedated by intraperitoneal injection of nembutal (125 μg/g).

### Cell lines

MDCK, MDCK.PIV5V and HEK293T cells were cultured in DMEM supplemented with 10% FCS, non-essential amino acids, 2 mM L-glutamine, 0.4 mM sodium-pyruvate, 100 U/ml penicillin and 0.1 mg/ml streptomycin at 37°C in 5% CO_2_. MDCK cells stably expressing the type I IFN antagonist Paramyxovirus Simian Virus 5 V protein (MDCK.PIV5V) were kindly provided by Dr. Rick Randall (University of St. Andrews, United Kingdom) [28, 29].

### Construction of the plasmid pHW-NS1(1–73)Dmd-GFP-NEP

The coding sequence of HAtag/Dmd/FMDV-2A was generated synthetically (Genscript) and cloned into the pcDNA3 vector using the restriction sites *Not*I and *Xba*I. The sequence coding for the first 73 amino acids of NS1 was amplified by PCR from the pHW198-NS plasmid [30] and cloned 5' of and in frame with the HA-tag using *Bam*HI and *Esp*EI. This NS(1–73)-HAtag/Dmd/FMDV-2A was then cloned into the pHW2000 plasmid using the *Bam*HI and *Mun*I restriction sites. The Quantum SuperGlo GFP coding sequence (derived from Qbiogene vector pQBI25-fc1) was cloned 3’ of and in frame with the FMDV-2A cleavage site using *Bgl*II and *Eco*RI. The PTV-1 2A cleavage site was fused to the NEP coding sequence (by fusion PCR) and cloned 3' of and in frame with the GFP coding sequence using the *Eco*RI and *Bst*EII restriction sites.

### Production of recombinant viruses

Recombinant viruses were rescued using the influenza A/Puerto Rico/8/34 based reverse genetics system [30]. To generate recombinant virus, 1 μg of each of the seven pHW-plasmids (pHW191-PB2, pHW192-PB1, pHW193-PA, pHW194-HA, pHW195-NP, pHW196-NA, pHW197-M) was transfected together with 1 μg of pHW198-NS (wild type PR8 virus) or 1 μg of pHW-NS1(1–73)Dmd-GFP-NEP (PR8-NS1(1–73)GFP virus) in a HEK293T/MDCK co-culture using calcium phosphate precipitation in Optimem. After 36 h, TPCK-treated trypsin (Sigma) was added to a final concentration of 2 μg/ml. After 72 h, the medium was collected. The virus in the medium was amplified on MDCK cells (wild type PR8) or MDCK.PIV5V cells (PR8-NS1(1–73)GFP virus) in serum-free cell culture medium in the presence of 2 μg/ml TPCK-treated trypsin (Sigma), and the viral titer was determined by plaque assay on MDCK cells.

### Influenza plaque assay

MDCK cells were seeded at 5 x 10^5^ cells per well in a six-well plate. The next day, the cells were infected with a ten-fold dilution series of the virus in serum-free medium, in a total volume of 1 ml. The inoculum was removed after 1 h of incubation at 37°C and replaced by an overlay of 0.6% Avicel RC-591 (FMC Biopolymer) in serum-free medium containing 2 μg/ml TPCK-treated trypsin (Sigma). After three days of incubation at 37°C, the overlay was removed and the cells were fixed with 4% paraformaldehyde and permeabilized with 0.2% Triton X-100. The cells were stained with an anti-M2e mouse monoclonal antibody and the plaques were visualized with TrueBlue peroxidase substrate (KPL). For the determination of the percentage of GFP-positive cells, the plaques were first detected with an anti-GFP antibody (A21311; Molecular probes) and subsequently with a monoclonal anti-M2e antibody or a polyclonal anti-influenza A serum.

### *In vitro* growth kinetics

MDCK cells (seeded at 4 x 10^6^ cells per 9-cm dish) were infected in duplicate with a MOI of 0.001 of wild type PR8 virus or PR8-NS1(1–73)GFP virus in a total volume of 5 ml. After 1 h of incubation, the inoculum was removed and replaced by 10 ml of serum-free medium containing 2 μg/ml TPCK-treated trypsin (Sigma). A 200 μl sample was taken at 0, 4, 8, 12, 24 and 48 h after removal of the inoculum. The viral titer in the samples was determined by TCID_50_ analysis.

### TCID_50_ analysis

MDCK cells were seeded at 2 x 10^4^ cells per well in a 96-well plate in complete DMEM. The next day, cells were washed once with serum-free medium and incubated with a ten-fold dilution series of sample in serum-free DMEM containing 2 μg/ml TPCK-treated trypsin (Sigma). After seven days, the presence of virus in each well was determined by hemagglutination with chicken red blood cells (1% solution). The TCID_50_ values were calculated by the method of Reed & Muench [31].

### Immunofluorescence

MDCK cells (seeded on glass coverslips at 2 x 10^4^ cells per well in a 24-well plate) were infected with a MOI of 1 of PR8-NS1(1–73)GFP virus or wild type PR8 virus. After 24 h, the cells were fixed with 1% paraformaldehyde, permeabilized with 0,2% Triton X-100, and stained with anti-influenza RNP (obtained from the NIH Biodefense and Emerging Infections Resources Repository, NIAID, NIH, NR-4282, polyclonal anti-influenza virus RNP, A/Scotland/840/74 (H3N2) (antiserum, Goat); diluted 1/2000). Alexa Fluor 555 donkey anti-goat IgG (Cat. No. A21432, Life Technologies Europe B.V.; diluted 1/800) was used as secondary antibody. Cell nuclei were visualized with Hoechst (Cat. No. H21492, Invitrogen; diluted 1/1000). Images were recorded with a confocal microscope (Leica Sp5 AOBS confocal system) using a 63x HCX PL Apo 1.4 oil immersion objective.

### Analysis of GFP expression by western blot

MDCK cells were seeded at 3 x 10^5^ cells per well in a 6-well plate and 24 h later infected with a MOI of 1 of PR8-NS1(1–73)GFP virus or wild type PR8 virus. Mock infected MDCK cells were included as negative control. After 24 h, the cells were lysed on ice for 30 min in 250 μl lysis buffer (50 mM Tris pH 8, 150 mM NaCl, 1% NP40, 5 mM EDTA with protease inhibitors (Complete; Roche Diagnostics N.V. Belgium)). Laemmli buffer containing β-mercaptoethanol was added and the sample was boiled for 10 min. The proteins were separated using SDS-PAGE, and GFP was visualized by western blot, using an anti-GFP antibody (A21311; Molecular probes), a monoclonal anti-HA antibody (clone 12CA5, 11583816001, Roche) or a monoclonal anti-NS1 antibody (sc-130568; Santa Cruz Biotechnology).

### Treatment and infection of mice

Eight-week-old female BALB/c mice were housed in specific pathogen free conditions with food and water *ad libitum*. To compare the lethality of PR8 and PR8-NS1(1–73)GFP viruses, mice were infected under mild isoflurane anesthesia with 1 x 10^3^ PFU of wild type PR8 and different doses of PR8-NS1(1–73)GFP virus (1 x 10^3^, 1 x 10^4^ or 1 x 10^5^ PFU). The bodyweight was monitored daily for 14 days.

To determine the viral titers in the lungs, mice (n = 8 per group) were anesthetized by ketamine/xylazine and infected with 1 x 10^3^ PFU of wild type PR8 or 1 x 10^3^ or 1 x 10^4^ PFU of PR8-NS1(1–73)GFP virus. Two or five days after infection, four mice from each group were terminally sedated by nembutal and the lungs were excised and homogenized in 1 ml of PBS with a Mixer Mill MM 200 for 8 min at an amplitude of 100. The lung homogenates were clarified by centrifugation for 10 min at 500 x g and the viral titers were determined by TCID_50_ analysis.

To determine the *in vivo* tropism and the effect of treatment with anti-M2e antibody and oseltamivir, BALB/c mice were treated as follows. One day before infection, mice in one group were passively immunized with 5 μg of anti-NBe (control antibody, directed against the NB protein of influenza B virus) or 5 μg anti-M2e monoclonal antibody. Antibodies were administered intranasally under isoflurane anesthesia. Another group of mice was treated for 6 consecutive days, starting one day before infection, with 25 mg/kg oseltamivir by gavage. The mice were infected, under mild isoflurane anesthesia, by intranasal administration of 1 x 10^4^ PFU of PR8-NS1(1–73)GFP virus diluted in 50 μl PBS. The control mice were infected with 1 x 10^3^ PFU of wild type PR8 virus and otherwise left untreated.

### Flow cytometry

On the indicated days after influenza virus infection, mice were terminally sedated with nembutal, and the lungs were removed and treated with collagenase and DNase. The lungs were subsequently forced through a 70 μM filter to produce single-cell suspensions. Erythrocytes were removed by lysis in NH_4_Cl red blood cell lysis buffer. The cells were incubated with anti-mouse CD16/CD32 antibody (FcBlock, BD) to avoid nonspecific immunostaining of immune cells and stained with anti-B220-Alexa Fluor 700, anti-CD11c-APC, anti-CD11b-APC-Cy7, anti-CD45-PerCP, anti-GR1-PE-Cy7, anti-CD3e-PE and anti-CD49b (DX5) V450 (all from BD) for 30 min. The number of GFP positive lung cells was determined on an LSR-II flow cytometer (BD, San Jose, CA) by analyzing surface expression of CD45, CD3e, B220, CD11b, CD11c, GR1, and DX5 using FACSDiva (BD) and FlowJo software (Treestar). The gating strategy used to define the different cell subsets are presented in S1 File.

### Deep sequencing analysis of PR8 and PR8-NS1(1–73)GFP virus

The presence of the wild type NS or the mutant NS1(1–73)GFP segment in the viral genome was confirmed by RT-PCR and sequence analysis. Total RNA was isolated from 2 x 10^5^ PFU of wild type PR8 or PR8-NS1(1–73)GFP virus with the high pure RNA isolation kit (Roche), and cDNA was synthesized with the Transcriptor first strand synthesis kit (Roche), both according to the instructions of the manufacturer. cDNA synthesis was performed with a primer specific for influenza A vRNA (nucleotides complementary to the conserved ends of the influenza A genomic segments are underlined in the primer sequence): CommonUni12G (GCCGGAGCTCTGCAGATATCAGCGAAAGCAGG). Next, all eight genomic segments were amplified in one reaction with Phusion High Fidelity polymerase (Thermo Scientific) using primers CommonUni12G and CommonUni13 (GCCGGAGCTCTGCAGATATCAGTAGAAACAAGG) [32, 33]. Before Illumina MiSeq sequencing, the quality of the DNA sample was evaluated using the Agilent High Sensitivity DNA kit (Agilent Technologies). A multiplexed paired-end sequencing library was generated on 0.5 ng of sample by using the NexteraXT DNA Sample Preparation Kit, which fragments and tags the sample DNA based on an engineered transposon, according to the instructions of the manufacturer. After the 2*250 bp MiSeq paired-end sequencing run, the data were base called and demultiplexed on the instrument. The downstream data analyses were performed on the resulting Illumina FASTQ files (Phred 64+ encoding) using CLC Genomics Workbench (Version 7.0.3) following the analysis pipeline as described in [32]. The adaptor contamination was removed and the sequencing reads were trimmed from both sides using the modified Mott trimming algorithm to reach a Q20 score. In addition, all ambiguous (N) bases, reads with a read length shorter than 50 nucleotides and reads with broken pairs resulting from this *in silico* trimming and filtering were removed. The resulting reads were aligned to the PR8-NS1(1–73)GFP reference genome using the following parameters: match = +1; mismatch = -2; insertion/deletion = -3; length fraction = 0.9; similarity fraction = 0.8; non-specific match handling = ignore. Sequence variants were called using all available sequencing data that covered each nucleotide at least 100 times and had a central base quality score of Q20 or greater. Further filtering of variants was performed with the following parameters: forward/reverse balance > 0.25; variant count > 10; frequency > 0.5% [32]. The error rate was determined after mapping the reads to the reference genome and calculated as the relative error for a single genome segment or the complete genome. The A-to-G variant at position 24 in the HA, NP, NA, M and NS segments introduced by the primer used for RT-PCR was not taken into account during the variant analysis or calculation of the error rate. The raw sequencing data can be found in the NCBI Sequence Read Archive with the accession numbers SRR1752132 for the PR8-NS1(1–73)GFP virus and SRR1766133 for the wild type PR8 virus.

### Passages on MDCK and MDCK.PIV5V cells

MDCK cells were infected with a MOI of 0.001 of PR8-NS1(1–73)GFP virus, and the supernatant was collected two days later. This supernatant was diluted 1/10^5^ and used to infected new MDCK cells for 2–3 days, after which the virus was diluted again 1/10^5^ and passaged to fresh MDCK cells. This protocol was used to passage the virus five times on MDCK cells. For the passages on MDCK.PIV5V cells, a five-fold dilution series of the PR8-NS1(1–73)GFP virus was used to infect the MDCK.PIV5V cells and every 2–3 days, the virus in the supernatant was quantified by hemagglutination. The virus in the well before the last well containing virus was then used at a 1/10 dilution for a subsequent passage on MDCK.PIV5V cells. Five passages were performed on these cells.

## Results

### Generation of recombinant influenza A virus expressing GFP

**Fig 1:**
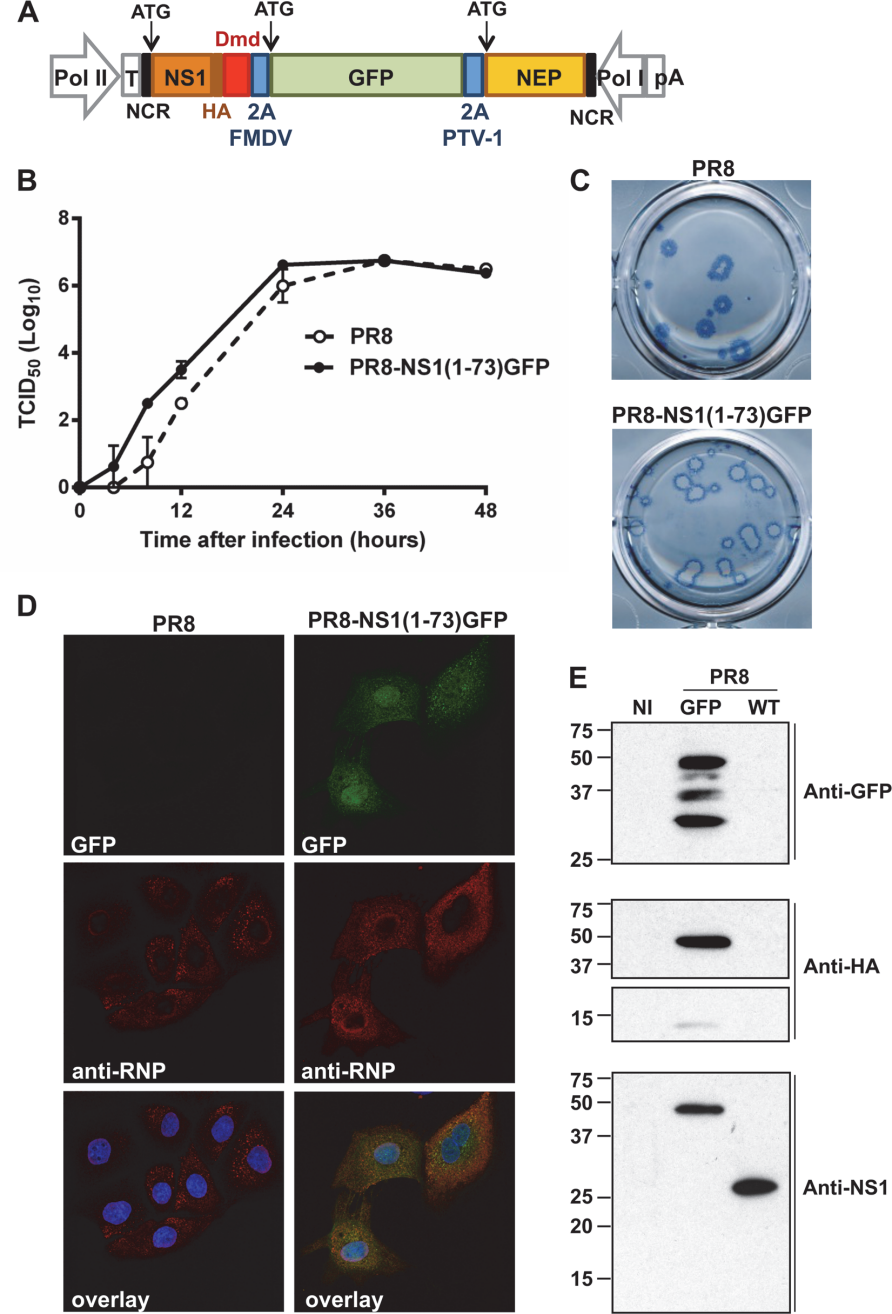
*In vitro* characterization of the PR8-NS1(1–73)GFP virus. (A) Schematic representation of the promoters and coding sequences of the pHW-NS1(1–73)Dmd-GFP-NEP plasmid used to generate the reporter GFP influenza virus. (B) Multi-cycle growth kinetics. MDCK cells were infected in duplicate with a MOI of 0.001 of wild type PR8 virus or PR8-NS1(1–73)GFP virus in the presence of TPCK-trypsin. At the indicated time points after infection, the viral titer in the supernatant (50 μl sample) was determined by TCID_50_ analysis. The graph shows the mean with the standard error of each data point. For the wild type PR8 virus, one of the duplicate samples of the 48 h time point was excluded due to technical failure. (C) Plaques of PR8 and PR8-NS1(1–73)GFP virus were visualized on day three after infection of MDCK cells by immunostaining with an M2e-specific monoclonal antibody. (D) Confocal microscopy analysis of MDCK cells infected with wild type PR8 or PR8-NS1(1–73)GFP virus (MOI 1). Twenty four hours after infection the cells were fixed and stained with anti-RNP (red; middle panel) and Hoechst (blue). The GFP signal is shown in green (top panel). An overlay of the three colors is shown in the bottom panel. (E) MDCK cells were infected with a MOI of 1 of PR8-NS1(1–73)GFP virus or wild type PR8 virus, or were not infected (NI). After 24 h, lysates were prepared and the proteins were visualized by western blotting and immune-detection with an anti-GFP (top), anti-HA (middle) or anti-NS1 (bottom) antibody. The panel of anti-HA is split in two parts, as different exposure times were used to reveal the protein bands.

Our aim was to generate a fit GFP expressing influenza A virus by engineering the NS gene segment (*i*.*e*. genomic RNA segment 8) while keeping the length of the resulting recombinant segment as short as possible. The NS gene segment of influenza viruses encodes two proteins: the type I IFN antagonist NS1 is translated from full-length mRNA, and the nuclear export protein (NEP) is translated from a spliced mRNA. Binding of NS1 to RNA is important for its biological function, including suppression of RIG-I activation [34]. The RNA binding and IFN antagonistic activities of NS1 are confined to the 73 amino acid N-terminal part of NS1 [35, 36]. Influenza viruses expressing C-terminally truncated NS1 variants are attenuated *in vivo*, but fusion of the N-terminal RNA-binding domain to a heterologous dimerization domain restores pathogenicity in mice [37]. We reasoned that it might be possible to increase the insertion capacity of the influenza A genome without compromising viral fitness and pathogenicity in laboratory mice, by retaining the coding information for the N-terminal 73 amino acids of NS1 fused at the C-terminus to the dimerization domain (Dmd) of the *Drosophila melanogaster* Ncd protein [37]. We therefore designed a tri-cistronic NS-derived gene segment with a single open reading frame comprising NS1(1–73)Dmd, GFP and NEP separated from each other by two different 2A self-processing sites. We used two different 2A peptide sequences to reduce the risk of recombination at these sites, as this could lead to the excision of the GFP coding information. A foot-and-mouth disease virus (FMDV) 2A auto processing site was inserted between NS1(1–73)Dmd and GFP, while the latter was separated from NEP by a porcine teschovirus-1 (PTV-1) 2A cleavage site (Fig. 1A). The FMDV 2A peptide was the first 2A cleavage site to be described, and has been used for many applications including the generation of recombinant influenza viruses [38–41]. In addition, the PTV-1 2A cleavage site has been shown to have a very high cleavage efficiency [42, 43]. Finally, an HA-tag was fused to NS1 to facilitate protein detection. This artificial NS segment was used to rescue an influenza virus expressing dimeric NS1(1–73), GFP and NEP in a PR8 virus genetic background [30]. This rescue was successful and we named the resulting virus PR8-NS1(1–73)GFP.

### *In vitro* characterization of the PR8-NS1(1–73)GFP virus

To assess whether truncation of the NS1 gene or the insertion of GFP affected viral fitness *in vitro*, we compared growth kinetics, plaque size and plaque morphology in MDCK cells with those of wild type PR8 virus. First, we used a multi-cycle replication assay on MDCK cells to compare the growth kinetics of PR8-NS1(1–73)GFP virus and wild type PR8 virus. We infected MDCK cells with a multiplicity of infection (MOI) of 0.001 and determined the virus titer in the supernatant at different time points after infection. The slope of the growth curve and the endpoint titer reached after two days were comparable for PR8-NS1(1–73)GFP and wild type PR8 virus, suggesting that the two recombinant viruses have similar growth kinetics (Fig. 1B; *P* > 0.05, paired t-test). A plaque assay showed that plaques of PR8-NS1(1–73)GFP virus were slightly larger and had a thinner M2-immunoreactive halo than those of the parental PR8 virus (Fig. 1C).

To test whether GFP was expressed in infected cells, we used fluorescence microscopy. MDCK cells were infected with PR8-NS1(1–73)GFP virus or wild type PR8 virus at a MOI of 1, and 24 h later the cells were monitored for GFP fluorescence and stained for viral ribonucleoprotein (RNP) expression. GFP and RNPs were co-expressed in PR8-NS1(1–73)GFP virus infected cells (Fig. 1D). While NP expression was largely confined to the cytoplasm at this time point after infection, GFP expression was visible in the cytoplasm as well as in the nucleus. Next, we determined if the three proteins encoded by the mutant NS segment are produced as individual proteins. Ideally, the separation of NS(1–73)Dmd, GFP and NEP by the 2A auto-processing sites should generate three individual proteins. We infected MDCK cells (MOI 1) with PR8 or PR8-NS1(1–73)GFP virus and performed western blot analysis to determine the extent of cleavage between these three proteins. Detection with an anti-GFP antibody revealed three major bands of approximately 50 kDa, 37 kDa and 27 kDa (Fig. 1E). The higher band most likely corresponds to the uncleaved polyprotein (predicted size of 58.4 kDa) and the band of 27 kDa to GFP. The middle band likely corresponds to the NS1Dmd-GFP (predicted size of 43.9 kDa) or to the GFP-NEP fusion protein (predicted size of 43.2 kDa). Detection with a monoclonal antibody directed to the HA-tag which was introduced at the C-terminus of the altered NS1 part, also revealed the uncleaved polyprotein (50 kDa) and a minor band of approximately 15 kDa, most likely corresponding to the cleaved NS1(1–73)Dmd protein. Detection with an anti-NS1 monoclonal antibody revealed only a band of 50 kDa in lysates of PR8-NS1(1–73)GFP infected cells, corresponding to uncleaved precursor protein and a band of approximately 25 kDa corresponding to full-length NS1 in PR8 infected cells (Fig. 1E). Since the anti-HA and anti-NS1 antibodies did not reveal the 37 kDa band that was detectable with anti-GFP, this band most probably corresponds to the GFP-NEP fusion protein (Fig. 1E). This western blot analysis indicates that the cleavage at the 2A cleavage sites is incomplete. Taken together, PR8-NS1(1–73)GFP virus replicates as efficiently as wild type PR8 virus *in vitro*, and infection of cells with PR8-NS1(1–73)GFP virus results in GFP expression and green fluorescence, though processing at the introduced 2A sites is incomplete.

### PR8-NS1(1–73)GFP virus is pathogenic in mice

**Fig 2:**
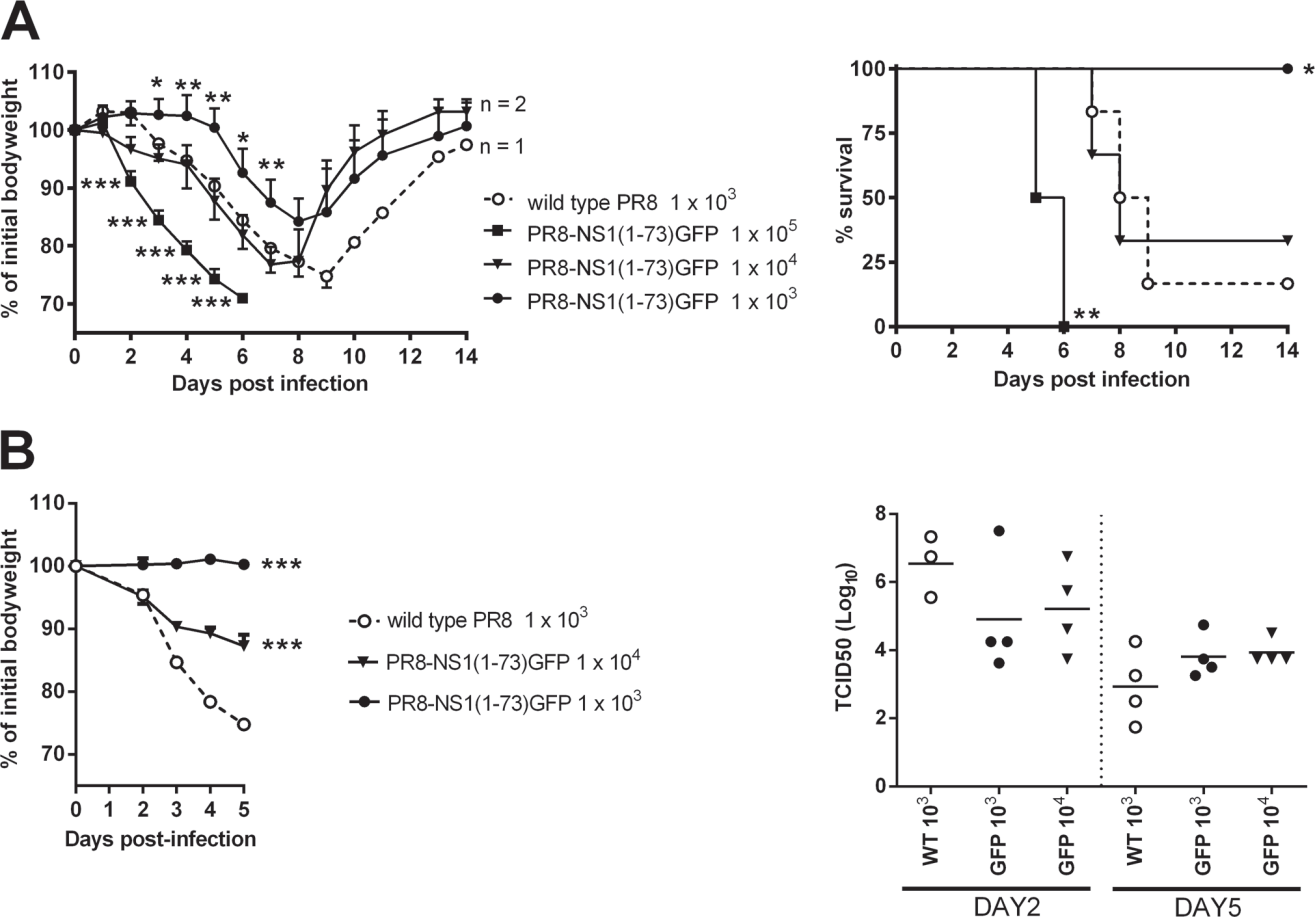
PR8-NS1(1–73)GFP virus is pathogenic in mice. (A) BALB/c mice (n = 6 per group) were inoculated with 1 x 10^3^, 1 x 10^4^ or 1 x 10^5^ PFU of PR8-NS1(1–73)GFP virus or 1 x 10^3^ PFU of wild type PR8 virus. Body weight (relative to initial body weight on day 0) and survival were monitored for 14 days. Error bars represent the standard deviation. The body weight of mice infected with 1 x 10^3^ or 1 x 10^5^ PFU of PR8-NS1(1–73)GFP virus was significantly different on day 3–7 or day 2–6, respectively, from mice infected with 1 x 10^3^ PFU of wild type PR8 virus (one-way ANOVA with a Tukey’s multiple comparison test, * *P* < 0.05, ** *P* < 0.01, *** *P* < 0.001). The survival curves of mice infected with 1 x 10^3^ or 1 x 10^5^ PFU of PR8-NS1(-173)GFP virus were significantly different from mice infected with 1 x 10^3^ PFU of wild type PR8 virus (log-rank test, * *P* < 0.05, ** *P* < 0.01) (B) BALB/c mice (n = 8 per group) were inoculated with 1 x 10^3^ or 1 x 10^4^ PFU of PR8-NS1(1–73)GFP virus or 1 x 10^3^ PFU of wild type PR8 virus. Body weight (relative to initial body weight on day 0) was monitored for 5 days. Data points represent averages and error bars represent the standard deviation. Body weight of mice infected with PR8-NS1(1–73)GFP virus was significantly different from those infected with wild type PR8 virus (two-way ANOVA with a Tukey’s multiple comparison test, *** *P* < 0.001). The virus titer in the lungs was assessed on day 2 and day 5 by TCID_50_ analysis of the lung homogenates (50 μl sample). For the titers on day two in the wild type PR8 infected group, one value was excluded due to technical failure. Differences in viral titers on day 2 or day 5 were not statistically significant (*P* > 0.05, one-way ANOVA, with a Tukey’s multiple comparison test).

Previous work showed that WSN virus expressing the N-terminal 73 amino acid residues of NS1 fused to a heterologous dimerization domain was pathogenic in mice [37]. The PR8-NS1(1–73)GFP virus described here was built on the genetic background of PR8 virus, which is well adapted to mice. However, the modifications in vRNA segment 8 increased its size from 890 (wild type) to 1600 nucleotides. This increase in size combined with the loss of the C-terminal domain of NS1 and loss of the mRNA splicing-dependent control of NEP expression might compromise the *in vivo* fitness of PR8-NS1(1–73)GFP [44, 45]. To test this, BALB/c mice (n = 6 per group) were inoculated intranasally with various doses (1 x 10^3^–1 x 10^5^ PFU) of PR8-NS1(1–73)GFP virus. Body weight was measured daily for 14 days, and weight loss was compared with a group of mice that received 1 x 10^3^ PFU of wild type PR8 virus. All mice lost significant body weight (Fig. 2A). Compared to wild type PR8 virus, a tenfold higher inoculum dose of PR8-NS1(1–73)GFP virus was needed to reach comparable morbidity (Fig. 2A). Five out of six mice infected with 1 x 10^3^ PFU of wild type PR8 virus died by day 9. Four out of six mice died when given 1 x 10^4^ PFU of NS1(1–73)GFP virus (Fig. 2A). In a second mouse experiment, we compared the replication of wild type PR8 and PR8-NS1(1–73)GFP virus. For this, we inoculated BALB/c mice (n = 8 per group) intranasally with 1 x 10^3^ PFU of wild type PR8 virus or 1 x 10^3^ or 1 x 10^4^ PFU of PR8-NS1(1–73)GFP virus. Body weight was measured daily and the viral titers in the lungs were determined on day two and day five post infection (Fig. 2B). Mice infected with wild type PR8 virus reached higher titers on day two than mice infected with PR8-NS1(1–73)GFP virus. However, these differences were not statistically significant (*P* > 0.05, one-way ANOVA, with a Tukey’s multiple comparison test). On the other hand, titers of PR8 virus were slightly lower than those of PR8-NS1(1–73)GFP virus on day five, but again this difference did not reach statistical significance (Fig. 2B). Taken together, these results indicate that PR8-NS1(1–73)GFP virus replicates efficiently in the lungs of BALB/c mice causing substantial morbidity and mortality, but is attenuated compared to wild type PR8.

### Kinetics and *in vivo* cell tropism of the PR8-NS1(1–73)GFP virus

**Fig 3:**
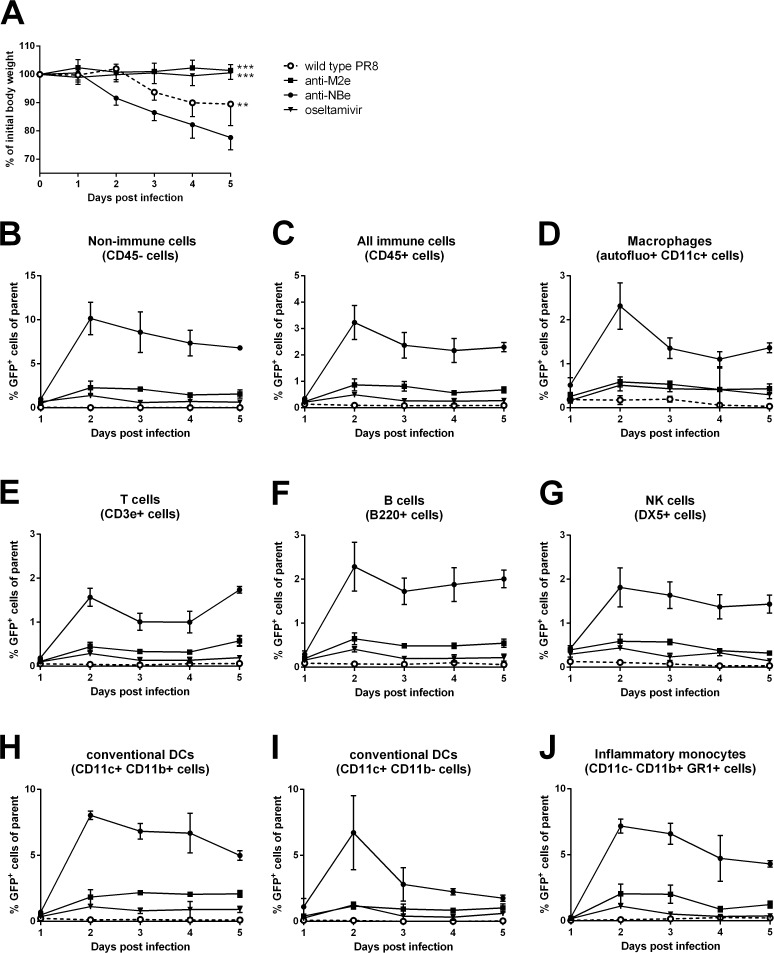
Kinetics and *in vivo* cell tropism of the PR8-NS1(1–73)GFP virus in mice. BALB/c mice (n = 19 per group) were infected with 1 x 10^4^ PFU of PR8-NS1(1–73)GFP or 1 x 10^3^ PFU of wild type PR8 virus. Each day, three mice from each group were euthanized. The lungs were isolated and the number of GFP positive cells was determined using multicolor flow cytometry. (A) Body weight was monitored for five days. Average body weight relative to initial weight on day 0 (n = 7, except for wild type n = 6). Body weights of mice treated with anti-M2e or oseltamivir were significantly different from those of mice treated with anti-NBe (** *P* < 0.01, *** *P* < 0.001); two-way ANOVA with a Tukey’s multiple comparison test. (B-J) GFP expression was analyzed in non-immune (CD45^-^) cells (B), and in immune (CD45^+^) cells (C), including macrophages (D), T cells (E), B cells (F), NK cells (G), dendritic cells (H-I) and inflammatory monocytes (J). The error bars represent the standard deviation. This graph is representative of two independent experiments.

Having established that PR8-NS1(1–73)GFP was pathogenic in mice, we decided to determine the *in vivo* infection kinetics and cell tropism of the PR8-NS1(1–73)GFP influenza virus. We performed this analysis in the context of matrix protein 2 ectodomain (M2e) based immune protection or oseltamivir treatment [46]. Protection elicited by immunization with M2e-fusion constructs is largely based on IgG and requires a functional Fcγ Receptor compartment and alveolar macrophages [47, 48]. Moreover, M2e-based protection is “infection permissive” and does not seem to interfere with the extent of CD8^+^ T cell responses induced upon viral challenge [49, 50]. Three groups of BALB/c mice were infected with 1 x 10^4^ PFU of PR8-NS1(1–73)GFP virus per mouse. M2e-specific immune protection was provided by intranasal administration of 5 μg of M2e-specific mouse monoclonal antibody to one group of mice, one day before challenge with PR8-NS1(1–73)GFP virus. As a negative control for this setup, a second group received 5 μg of an influenza B virus specific monoclonal antibody directed against the ectodomain of NB, one day before challenge. Mice in the third PR8-NS1(1–73)GFP infected group were treated daily with 25 mg/kg of oseltamivir, administered orally starting one day before challenge and for 6 subsequent days. As a negative control for GFP expression, one group of BALB/c mice was infected with 1 x 10^3^ PFU of wild type PR8 virus. A single administration of anti-M2e antibody protected mice from weight loss following PR8-NS1(1–73)GFP infection as efficiently as daily administration of oseltamivir (Fig. 3A). In contrast, animals receiving the negative control antibody gradually lost weight following infection with PR8-NS1(1–73)GFP virus (Fig. 3A). To determine the progress of GFP fluorescence in different cell types in the lungs, three mice from each group were sacrificed on five consecutive days, starting the first day after infection. Using multicolor flow cytometry to trace GFP expression in different cell types of the lungs (S1 and S2 Files), we detected GFP positive cells in the lungs of mice infected with PR8-NS1(1–73)GFP, but not in mice infected with wild type PR8 virus (Fig. 3B-J and S2 File). The primary host cells of influenza A virus are respiratory epithelial cells, but hematopoietic cells can also be infected by this virus, although this is not always a productive infection [51–55]. In non-hematopoietic cells (CD45^-^) of the control treated group, the percentage of GFP-expressing cells peaked on day 2 and then decreased slightly during the five days of analysis (Fig. 3B). For all lung hematopoietic cell types that were analyzed, a similar peak of GFP positivity was observed for the control-treated group two days after infection (Fig. 3C-J and S2 File). The highest level of GFP expression was detected in conventional dendritic cells (cDCs), which were defined as low autofluorescent CD45^+^ cells with CD11c surface expression. Two days after infection, between 5% and 10% of these cDCs were GFP-positive (Fig. 3H-I). For the CD11b^+^ cDCs (this population also includes the monocyte-derived DCs), GFP expression only decreased slightly after day 2 (Fig. 3H). In contrast, GFP expression decreased rapidly in the CD11b^-^ cDCs (Fig. 3I). It is noteworthy that the CD11b^-^ cDC population also includes the migratory CD103^+^ cDCs, which are important for presentation of viral antigen to T cells. Approximately two percent of the CD45^+^ CD11c^+^ autofluorescent population (mainly macrophages) were also GFP positive on day two (Fig. 3D). Of the CD11c^-^ CD11b^+^ GR1^+^ population (inflammatory monocytes and neutrophils), nearly 8% was GFP-positive on day two and GFP expression only decreased slightly over the next three days, similar to the CD11b^+^ cDCs (Fig. 3J). Finally, 1–2% of the B, T and NK cells became GFP positive, suggesting that they are susceptible to influenza A virus infection (Fig. 3E-G). Treatment with anti-M2e IgG2a or with oseltamivir resulted in a strong decrease in the percentage of GFP-expressing cells for all cell types analyzed. This suggests that both treatments can inhibit infection of epithelial cells and thereby indirectly reduce GFP levels in antigen presenting cells, and/or inhibit the infection of immune cells themselves.

### Genetic stability of PR8-NS1(1–73)GFP virus

**Fig 4:**
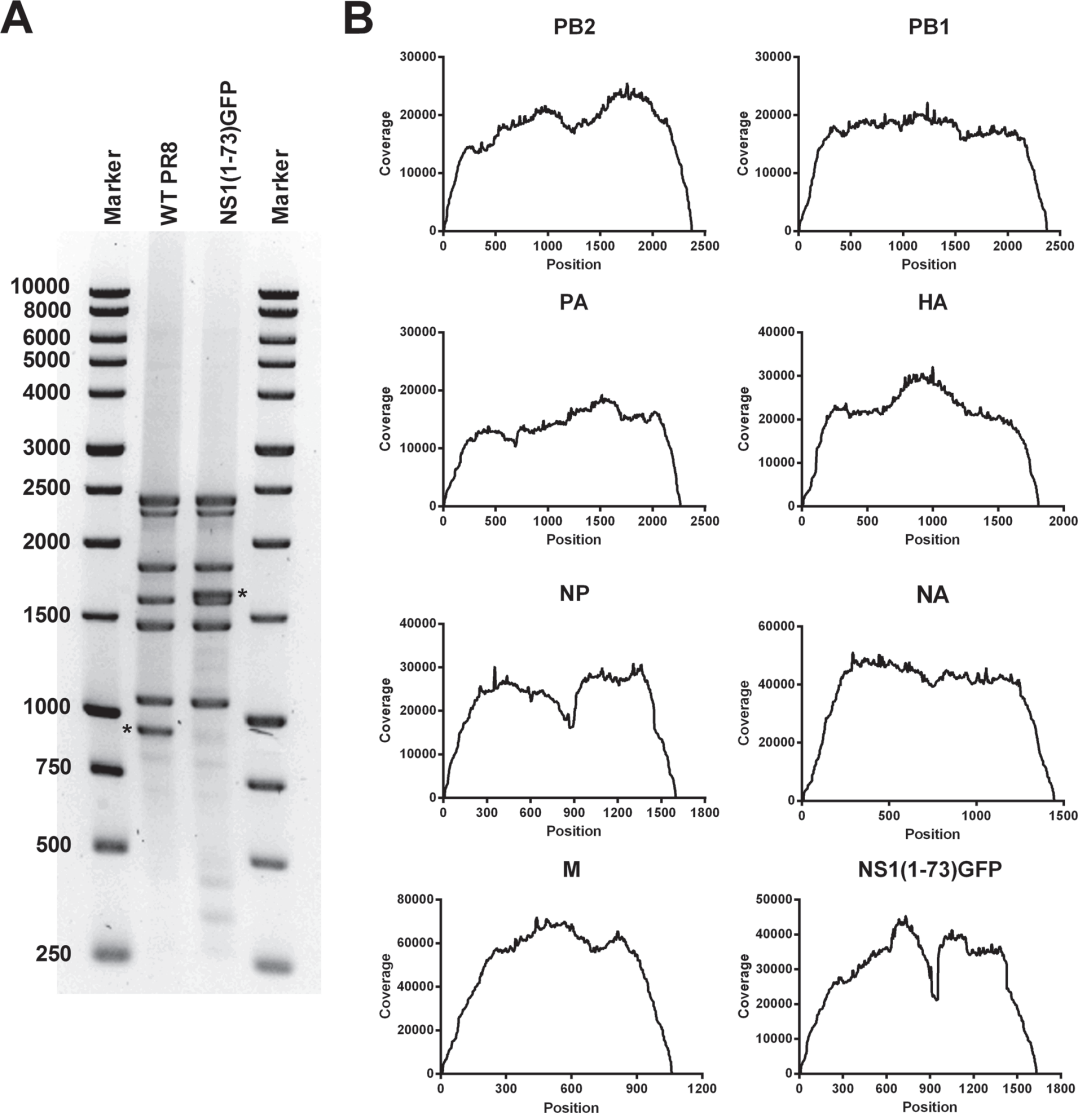
Nucleotide sequence analysis of PR8-NS1(1–73)GFP virus. (A) Electrophoretic analysis of RT-PCR products generated from viral RNA extracted from virus particles of PR8 (lane 2) and PR8-NS1(1–73)GFP (lane 3). The asterisk indicates the RT-PCR product derived from wild type NS or the engineered NS1(1–73)GFP gene segment. (B) Sequence coverage for the different genome segments of the PR8-NS1(1–73)GFP virus stock as determined by Illumina MiSeq sequencing and CLC genomics version 7.0.3 workbench data processing. The obtained sequences were mapped to the reference genome based on the plasmids used to generate the virus.

We analyzed the genetic composition of PR8-NS1(1–73)GFP virus, reasoning that potential loss of GFP expression would represent a competitive advantage over the parental virus. Electrophoretic analysis of RT-PCR amplified virion-associated gene segments revealed eight distinct bands for wild type PR8 virus, with the slowest migrating bands corresponding to a doublet of PB1 and PB2 (Fig. 4A, lane 2). A similar analysis of the RT-PCR amplified vRNA segments of PR8-NS1(1–73)GFP virus revealed that the band corresponding to the wild type NS segment was absent. Instead, we detected an additional band of 1600 base pairs migrating slightly slower than the RT-PCR product corresponding to the NP-coding segment 5 (Fig. 4A, lane 3). This confirmed the presence of the longer NS1(1–73)GFP gene segment in this virus. The PCR products were subsequentlty subjected to Illumina Miseq sequencing analysis to assess the genetic homogeneity of the PR8-NS1(1–73)GFP virus stock. Sequence coverage ranged from approximately 10,000 to 70,000 for each nucleotide across all genome segments, except for the 5' and 3' terminal nucleotides (Fig. 4B). This reduced coverage at the ends of a linear genomic fragment is inherent to the transposon-based fragmentation [56]. Comparison of the obtained sequences to the reference genome, based on the eight plasmids from which the virus was derived, showed that all viral segments contained mutations with a frequency > 0.5%. These mutations include single nucleotide variations (SNV), insertions and deletions (S3 File). The HA segment had the largest number of mutations (18 mutations) whereas the NS1(1–73)GFP segment showed only one mutation, present in the NEP ORF. To estimate if the PR8-NS1(1–73)GFP virus has a higher mutation frequency than the parental plasmid-derived PR8 virus, we compared the total error rate of both viral genomes (Table 1). We note that the sequencing data and *in silico* analysis pipeline for the parental PR8 virus are described in Van den Hoecke *et al*. [32]. Deep sequencing analysis of both viruses revealed an error rate of 0.13%, indicating that the two virus stocks have a similar level of homogeneity (Table 1). In addition, pairwise comparison of the percentage of errors in each viral genome segment, showed no difference between the two viruses, suggesting that wild type and NS1(1–73)GFP segments are equally stable (Table 1).

**Table 1:** Error rate^a^ (%) of the PR8 viruses.

	WT PR8 virus	PR8-NS1(1–73)GFP virus
**PB2 segment**	0.12%	0.13%
**PB1 segment**	0.12%	0.16%
**PA segment**	0.13%	0.13%
**HA segment**	0.21%	0.18%
**NP segment**	0.14%	0.13%
**NA segment**	0.12%	0.12%
**M segment**	0.10%	0.11%
**NS segment**	0.13%	0.13%
**Complete genome**	0.13%	0.13%

^a^ This error rate is the sum of errors introduced by the viral RNA-dependent RNA polymerase, by RT-PCR and by Illumina MiSeq sequencing.

It has been reported that GFP positivity may be lost during multiple rounds of replication of GFP-expressing influenza A viruses [16, 23]. To assess the genetic stability of the PR8-NS1(1–73)GFP virus *in vitro*, we performed a multi-cycle replication assay on MDCK cells. MDCK cells were infected with a MOI of 0.001 and the amount of newly produced GFP-positive viral particles was determined by plaque assay 24 h and 48 h post infection (Table 2). This experiment showed that most of the plaques remained GFP-positive (97.0% ± 2.6 at 24 h and 100% ± 0 at 48 h). Next, we passaged the PR8-NS1(1–73)GFP virus several times in MDCK cells. After each round of replication, during which virus was allowed to replicate for 2–3 days, a sample was taken from the cell culture medium. Because serial passages on MDCK cells could result in a mixture of virus, of which a certain percentage might have lost GFP expression, we visualized GFP expression in individual plaques originating from each *in vitro* passage of PR8-NS1(1–73)GFP virus. This revealed that the number of GFP-positive viruses decreased starting from passage 3 (Table 2). In addition to stabilizing the NS1 dimer, the C-terminal part of NS1 also assists the N-terminal domain to block interferon production by inhibiting TRIM25-mediated RIG-I ubiquitination [57]. Since this part of NS1 is not present in the PR8-NS1(1–73)GFP virus, it is possible that during multiple passages in MDCK cells the IFN response gradually increases and favors the replication of progeny virus with a truncated NS1 segment, *i*.*e*. with a deletion in the GFP ORF. We therefore passaged the virus in MDCK.PIV5V cells. These cells stably express the V protein of parainfluenza virus 5 (PIV5), an inhibitor of the host's interferon response, and we hypothesized that this protein could functionally complement the truncated NS1 protein. We found that PR8-NS1(1–73)GFP virus remained 100% GFP-positive for at least five passages in these cells (Table 2). Finally, we also determined the *in vivo* stability of the PR8-NS1(1–73)GFP virus. We infected BALB/c mice (n = 6 per group) with 1 x 10^4^ PFU of PR8-NS1(1–73)GFP virus and isolated the lungs five days later. We performed a plaque assay on the lung homogenates to determine the percentage of GFP-positive plaques among the total number of plaques. This showed that most of the viruses (96.4% ± 5.19%) that were isolated from the lungs five days after infection still expressed GFP (Table 2). Taken together, these results show that our PR8-NS1(1–73)GFP virus is relatively stable, both *in vitro* and *in vivo*.

**Table 2:** *In vitro* and *in vivo* stability of the PR8-NS1(1–73)GFP virus.

	No of GFP^+^ plaques/ Total No of plaques^a^	Group average % GFP ^+^ (SD)
**Stock (passage 0)**	6/6, 10/10, 5/6	94.4 (9.2)
**Multicycle growth—0 h**	8/8	100.0
**Multicycle growth—24 h**	22/23, 20/21, 21/21	97.0 (2.6)
**Multicycle growth—48 h**	11/11, 12/12, 4/4	100.0 (0)
**MDCK passage 1**	24/24	100
**MDCK passage 2**	10/10	100
**MDCK passage 3**	3/8	37.5
**MDCK passage 4**	3/7	42.9
**MDCK passage 5**	4/17	23.5
**MDCK.PIV5V passage 5**	16/16, 17/17, 10/10, 13/13	100.0 (0)
**Lung homogenates** ^**b**^	28/29, 20/20, 13/15,	96.4 (5.2)
	18/18, 20/21, 13/13	

^a^ To determine the percentage of GFP-positive cells, the plaques were first stained with an anti-GFP antibody and subsequently with a monoclonal anti-M2e antibody or a polyclonal anti-influenza A serum.

^b^ BALB/c mice were infected with 1 x 10^4^ PFU of PR8-NS1(1–73)GFP virus and lung homogenates were prepared on day five after infection.

## Discussion

In this study, we describe the generation of a replication competent GFP-expressing influenza A virus that has similar *in vitro* replication kinetics as wild type PR8 virus, and is only slightly attenuated *in vivo*. We inserted the GFP-coding sequence in the NS segment between NS1 and NEP. Our strategy resembles that of Manicassamy *et al*., who reported that inserting GFP in segment 8 of PR8 virus and leaving the NS1 and NES ORFs intact resulted in a GFP reporter virus that was about 100-fold attenuated compared to wild type virus [16]. We adapted this strategy in an attempt to generate a GFP-expressing virus that has a higher *in vivo* pathogenicity and stability. Firstly, to avoid the expression of an NS1-fusion protein, we designed a tri-cistronic NS segment containing NS1(1–73)Dmd, GFP and NEP separated by two different 2A auto-cleavage sites. Although this strategy should theoretically result in three separate proteins, western blot analysis of infected cell lysates showed that cleavage was only partial, which might have contributed to the *in vivo* attenuation of the PR8-NS1(1–73)GFP virus. Therefore, it could be interesting to also evaluate the cleavage efficiency of the two 2A sites *in vivo*. Polyprotein processing could be improved by changing or optimizing one or both of the 2A auto-proteolytic cleavage sites, as not all 2A sites show the same cleavage efficiency [42]. Nonetheless, the efficiency of different 2A sites will have to be determined empirically, as these may differ depending of the context. Secondly, to reduce the size of the NS segment, we used a truncated NS1 protein, which generally results in *in vivo* attenuation of the virus [58]. However, part of this attenuation can be reversed by adding a heterologous dimerization domain to the truncated NS1, which improves the stability of the dimeric NS1 protein [37].

Reporter gene expressing viruses ideally have a high genetic stability. This is difficult to accomplish with engineered influenza viruses. For example, Manicassamy *et al*. reported that in an *in vitro* multi-cycle replication assay already after 12h, 5–10% of the virus was no longer GFP-positive [16]. In addition, they observed that 5–30% of the virus in the lungs of mice was GFP-negative. Similarly, Kittel *et al*. reported the rapid appearance of NS-GFP deletion mutants after passage on MDCK cells, with complete loss of GFP positivity after several passages [23]. These GFP-negative viruses probably arise due to the selective advantage that viruses with truncations in the GFP sequence have over the parental virus. Deep sequencing analysis showed that the frequency of mutations in PR8-NS1(1–73)GFP virus stock was comparable to that in the parental PR8 virus (Table 1). We noticed a sequence coverage dip in the NP segment of wild type and PR8-NS1(1–73)GFP virus. A similar dip was present in the engineered NS1(1–73)GFP segment, corresponding to the middle of the GFP sequence. This reduced coverage can be explained by different mechanisms. A high mutation rate in this region could result in sequencing reads that cannot be mapped to the reference genome, resulting in a lower coverage. To assess this possibility, we compared the number of unmapped bases of both virus genomes. This revealed that 0.76% of bases derived from the wild type PR8 virus, and 0.64% of bases derived from the PR8-NS1(1–73)GFP virus could not be mapped to the respective reference genomes. This low percentage suggests that the coverage dips in the NP and NS-GFP segment do not result from an unusually high number of unmappable reads due to the presence of deletions or an excessive number of mutations. Alternatively, the reduced coverage in NP and GFP could be due to target sequence bias of the transposase that is used for fragmentation of the RT-PCR products or differences in GC-content [59].

To complement the sequence analysis, we analyzed the stability of the PR8-NS1(1–73)GFP virus by determining the number of GFP positive plaques after virus replication. These experiments showed that GFP expression was stable during the time course of a single experiment, whether *in vitro* or *in vivo* (Table 2). As the virus appears to be very stable when propagated or passaged in MDCK.PIV5V cells, we propose to use these cells to propagate influenza viruses with an engineered NS segment. Taken together, our results show that the PR8-NS1(1–73)GFP virus is genetically and phenotypically homogenous. Furthermore this virus is virulent in mice with an LD_50_ corresponding to approximately 1 x 10^4^ PFU.

Recombinant influenza viruses with a luciferase reporter in the PB2 [15], PA [18] or NA [17] segment have been described. These viruses appear to be stable and suitable for *in vivo* imaging of influenza replication and spread. However, viruses expressing luciferase are less suited to monitor viral antigen in different cell types by multicolor flow cytometry, which is more straightforward when a GFP reporter is used. We used PR8-NS1(1–73)GFP virus to study the cell tropism of PR8 virus in the lungs of infected mice. Non-immune cells (CD45^-^) were the major cell type that became GFP-positive, with a peak two days after PR8-NS1(1–73)GFP virus inoculation. This is not surprising, since epithelial cells are the primary targets of influenza viruses [60]. In addition, some immune cell types, such as cDCs, became up to 10% GFP positive, which corresponds to the results reported by Manicassamy *et al*. [16]. For most cell populations, GFP expression peaked on day two and decreased gradually over time. However, for the CD11b^-^ cDCs, which include CD103^+^ DCs, GFP expression nearly reached baseline levels again on day three (Fig. 3). This rapid decline is most probably due to migration of these GFP^+^ antigen presenting cells to the lung-draining lymph nodes, where they present influenza antigens to naive T cells. Apart from DCs, other immune cells in the lungs like T cells, B cells and NK cells also became GFP positive (on average 1–2%), suggesting that these cells can be infected by influenza virus. Finally, macrophages, neutrophils and monocytes that became GFP-positive may have acquired the reporter protein by direct (abortive) infection or by phagocytosis.

Treatment of mice with anti-M2e IgG monoclonal antibody and with oseltamivir resulted in a strong decrease of the percentage of GFP-expressing cells for all cell types analyzed. It is surprising that there was no obvious difference between the two treatments, although they are entirely different molecules (virus antigen-specific monoclonal antibody that protects in an Fcγ Receptor-dependent way *versus* a small compound that blocks viral NA activity). Moreover, they were administered differently: anti-M2e monoclonal antibody was administered only once intranasally 24 h before challenge, whereas oseltamivir was administered daily by oral gavage. Pre-existing anti-M2e IgG, in concert with Fcγ Receptor-bearing lung residing alveolar macrophages, could eliminate the first wave of infected respiratory epithelial cells. Therefore, the resulting effect on virus replication and spread might be quite comparable to that of daily administered oseltamivir, which also would mainly hinder newly produced viruses from spreading further in the lungs. Taken together, our results show that this PR8-NS1(1–73)GFP virus can be used to assess the efficacy of anti-influenza treatments or vaccination. This system could be adapted in the future to harbor other reporter proteins, *e*.*g*. red fluorescent protein. It could also be used with other genetic backgrounds of influenza virus, including different subtypes and influenza B viruses.

## Supporting Information

S1 File Gating strategy for the identification of the different mouse lung cell populations. Identification of the different cell populations was based on forward scatter (FSC), side scatter (SSC), autofluorescence, and the expression level of the surface markers CD45, CD3e, B220, DX5, CD11b, CD11c and GR1. The graphs are derived from the analysis performed two days after infection in one mouse treated with 5 μg anti-NBe antibody and infected with 1 x 10^4^ PFU of PR8-NS1(1–73)GFP virus.
(TIF)

S2 File GFP expression in the different cell populations. Determination of the amount of GFP expressing cells (%) in the different cell populations, for each treatment, on day two after infection (one mouse per group is shown). WT: one mouse that was left untreated and infected with 1 x 10^3^ PFU of WT PR8 virus. The mice in the other groups were infected with 1 x 10^4^ PFU of PR8-NS1(1–73)GFP virus and treated with 5 μg of anti-NBe, 5 μg of anti-M2e or 25 mg/kg oseltamivir. cDCs: conventional dendritic cells.
(TIF)

S3 File Mutations present in the PR8-NS1(1–73)GFP virus stock. The sequences obtained by Illumina Miseq analysis were mapped to the reference genome, which was based on the eight plasmids that were used to generate the virus. Mutations were considered significant when they occurred at a frequency > 0.5% (cut-off for background errors caused by the RT-PCR reaction), had a Phred score > 20, had a forward/reverse balance > 0.25 and appeared > 10x independently. SNV: single nucleotide variant.
(XLSX)
