# Supplementary figures and images for: A GFP Expressing Influenza A Virus to Report In Vivo Tropism and Protection by a Matrix Protein 2 Ectodomain-Specific Monoclonal Antibody
Source: PLoS One. 2015 Mar 27;10(3):e0121491. doi: 10.1371/journal.pone.0121491 (PMC4376807; doi:10.1371/journal.pone.0121491)

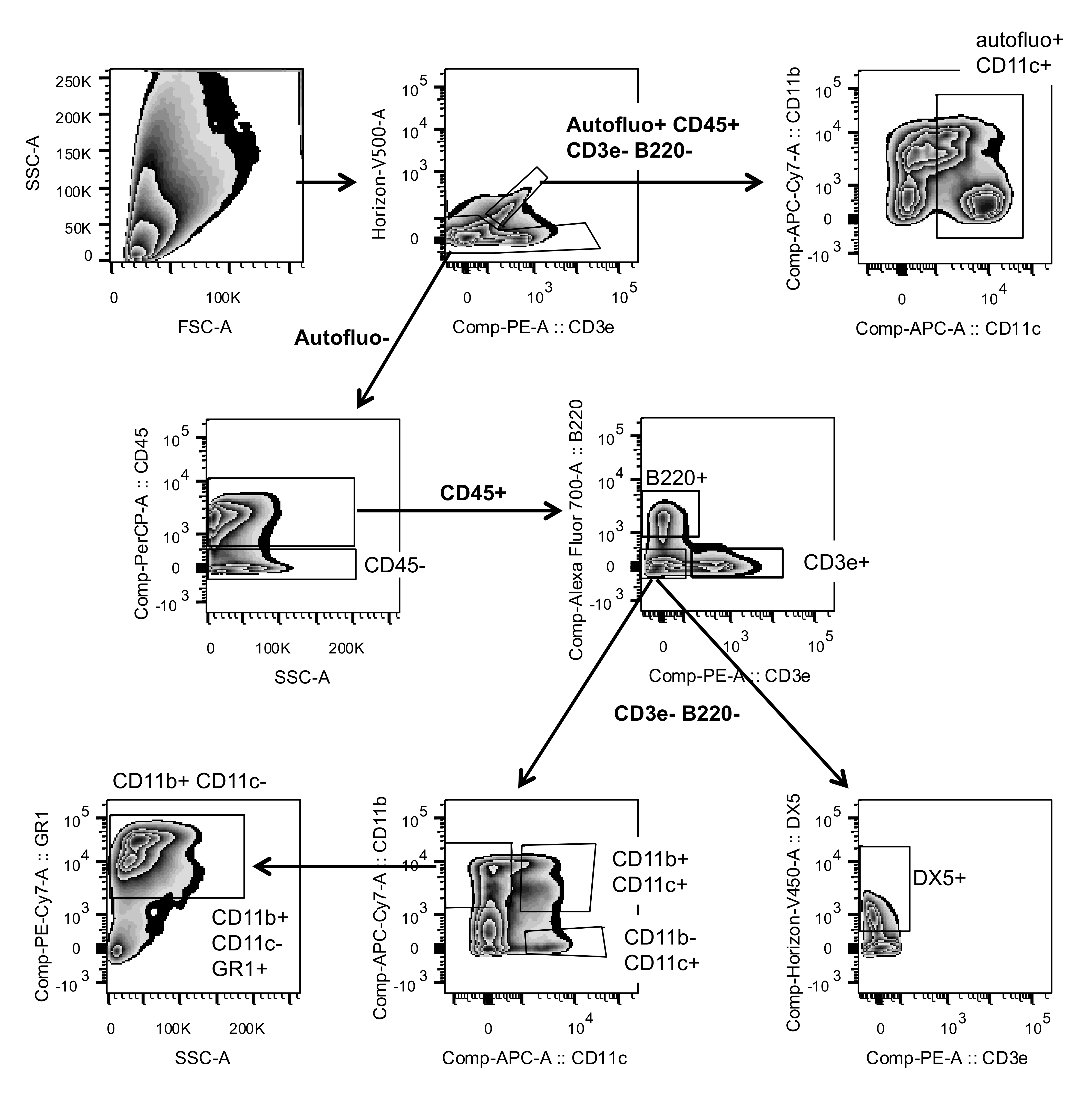

Supplement: S1 File — Identification of the different cell populations was based on forward scatter (FSC), side scatter (SSC), autofluorescence, and the expression level of the surface markers CD45, CD3e, B220, DX5, CD11b, CD11c and GR1. The graphs are derived from the analysis performed two days after infection in one mouse treated with 5 μg anti-NBe antibody and infected with 1 x 10^4^ PFU of PR8-NS1(1–73)GFP virus. (TIF) [file pone.0121491.s001.tif]

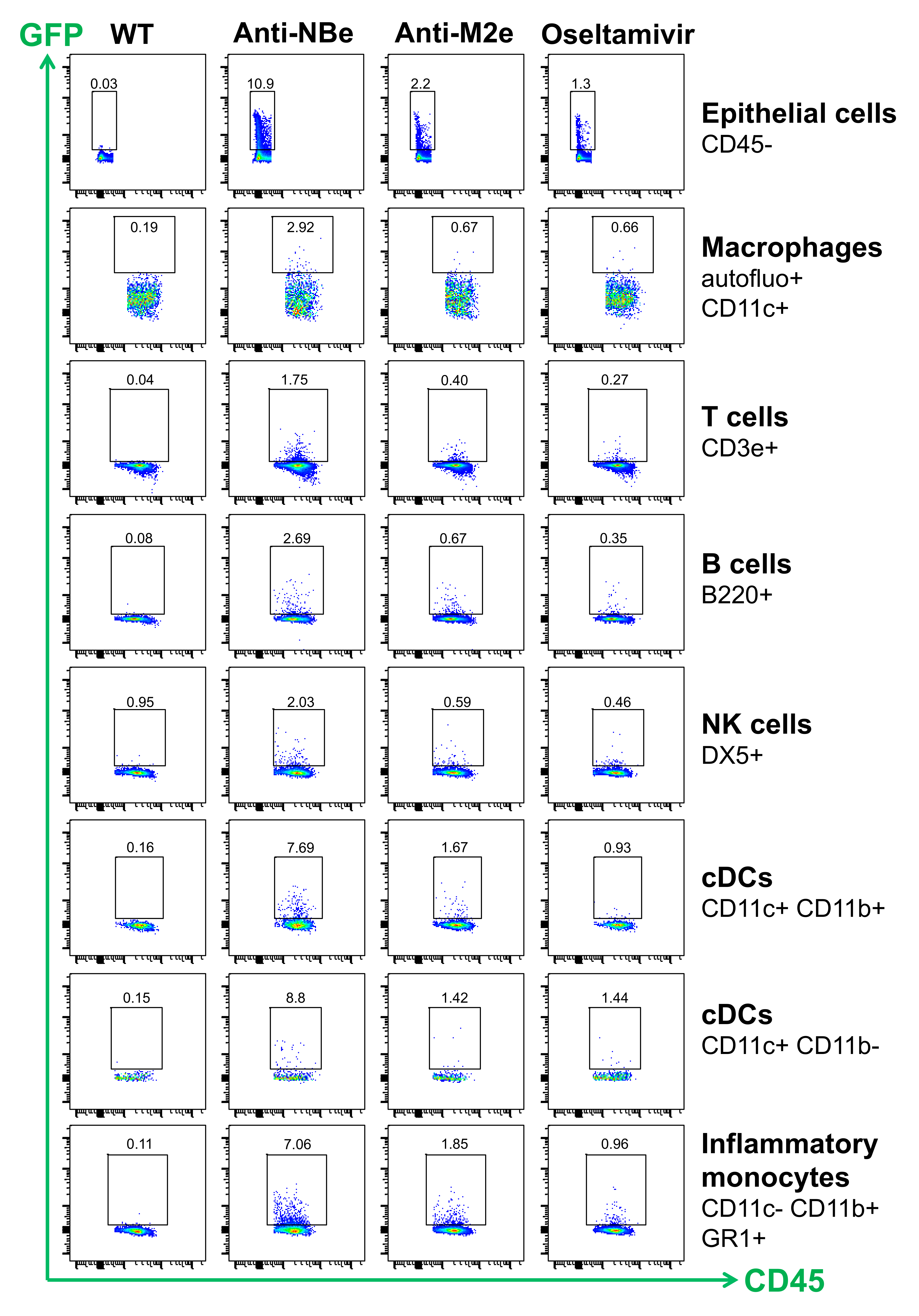

Supplement: S2 File — Determination of the amount of GFP expressing cells (%) in the different cell populations, for each treatment, on day two after infection (one mouse per group is shown). WT: one mouse that was left untreated and infected with 1 x 10^3^ PFU of WT PR8 virus. The mice in the other groups were infected with 1 x 10^4^ PFU of PR8-NS1(1–73)GFP virus and treated with 5 μg of anti-NBe, 5 μg of anti-M2e or 25 mg/kg oseltamivir. cDCs: conventional dendritic cells. (TIF) [file pone.0121491.s002.tif]
